# Evolution of Outcrossing in Experimental Populations of *Caenorhabditis elegans*

**DOI:** 10.1371/journal.pone.0035811

**Published:** 2012-04-23

**Authors:** Henrique Teotonio, Sara Carvalho, Diogo Manoel, Miguel Roque, Ivo M. Chelo

**Affiliations:** Instituto Gulbenkian de Ciência, Oeiras, Portugal; The University of Chicago, United States of America

## Abstract

*Caenorhabditis elegans* can reproduce exclusively by self-fertilization. Yet, males can be maintained in laboratory populations, a phenomenon that continues to puzzle biologists. In this study we evaluated the role of males in facilitating adaptation to novel environments. For this, we contrasted the evolution of a fitness component exclusive to outcrossing in experimental populations of different mating systems. We introgressed a modifier of outcrossing into a hybrid population derived from several wild isolates to transform the wild-type androdioecious mating system into a dioecious mating system. By genotyping 375 single-nucleotide polymorphisms we show that the two populations had similar standing genetic diversity available for adaptation, despite the occurrence of selection during their derivation. We then performed replicated experimental evolution under the two mating systems from starting conditions of either high or low levels of diversity, under defined environmental conditions of discrete non-overlapping generations, constant density at high population sizes (N = 10^4^), no obvious spatial structure and abundant food resources. During 100 generations measurements of sex ratios and male competitive performance showed: 1) adaptation to the novel environment; 2) directional selection on male frequency under androdioecy; 3) optimal outcrossing rates of 0.5 under androdioecy; 4) the existence of initial inbreeding depression; and finally 5) that the strength of directional selection on male competitive performance does not depend on male frequencies. Taken together, these results suggest that androdioecious males are maintained at intermediate frequencies because outcrossing is adaptive.

## Introduction

The androdioecious nematode *Caenorhabditis elegans* mostly reproduces by self-fertilization. Yet, males can be maintained within laboratory populations, a phenomenon that puzzles evolutionary biologists [1], [2], [3], [4], [5], [6], [7], [8], [9]. *C. elegans* predominant breeding mode in nature is self-fertilization but cross-fertilization can also occur between hermaphrodites and males [5], [6], [7]. The problem is that maintenance of males within populations imposes fitness costs if nothing else because with bi-parental reproduction the genetic representation of each individual in the next generation is halved relative to uni-parental reproduction such as self-fertilization (selfing) [1], [10]. In addition, there is outbreeding depression in *C. elegans* [11], [12], which suggests that outcrossing can lead to the disruption of beneficial gene combinations that can be easily maintained by selfing (cf. [13], [14]). Males should also impose ecological and physiological costs such as those involved in searching for mates or as a consequence of copulation [15], [16], [17], [18]. And finally, *C. elegans* hermaphrodites might be reluctant to mating, or unattractive to males [19], [20]. Considering such costs, for males to be maintained at higher frequencies than those expected from mutational input only they must disproportionally contribute to fitness when compared to selfing hermaphrodites [5], [7], [9].

Hypotheses on the maintenance of males in *C. elegans* all relate to the role of outcrossing on evolution since hermaphrodites cannot mate with each other (see [1] for a review). Relative to outcrossing, selfing reduces heterozygosity and increases linkage disequilibrium among polymorphisms. Consequently, effective segregation and recombination are reduced and thus also the prospect for novel genotypes to appear and be selected upon [21], [22], [23]. For instance, with segregation of partially-dominant or recessive deleterious alleles, male maintenance is favored because it mitigates inbreeding depression [1], [5], [24], [25]. In populations without genetic diversity however, selfing is expected to be more efficient at purging new deleterious alleles than outcrossing since more homozygotes are readily produced [26], [27], [28]. In *C. elegans* inbreeding depression should not however explain male maintenance because crosses among wild isolates have failed to show it [11], [29], and males have only a minor role in buffering partially-dominant deleterious mutations in laboratory populations [30], [31] (but see [2]).

In the presence of standing genetic diversity outcrossing can facilitate adaptation to novel environments [2], [5], [21]. This occurs when the additive fitness variance of a population is predominantly determined by phenotypes exclusively involved in outcrossing (e.g., male reproductive success) than those that are unique to selfing. Otherwise, an evolutionary conflict between selfing and outcrossing would arise and adaptation could fail. Analogously in the face of mutational input only, when compared to selfing, outcrossing can accelerate the combination of beneficial alleles from different loci, increase the frequency of overdominant alleles, and reduce hitch-hiking effects between deleterious and beneficial alleles that might constrain adaptation [14], [32], [33]. Several studies in *C. elegans* indirectly support such population genetic mechanisms. For instance, wild isolates show considerable quantitative genetic differentiation in male phenotypes, including male reproductive success [4], [34], [35], male sperm morphology [35], [36], and rates of male loss in laboratory populations [4], [31], [37]. In addition, laboratory populations consistently favor the maintenance of males for periods of up to 60 generations when in novel environments [1], [2], [3], [38]. Also, it has been shown that the strength of natural selection (possibly sexual selection among males) can determine the evolution of male sperm size [36] and the extent of adaptation [2].

Few studies have tested for the adaptive consequences of outcrossing by directly observing correlated changes with outcrossing-specific fitness components while at the same time controlling for standing genetic diversity (cf. [2], [36], [38]). Consequently, it is still poorly understood whether males might be maintained in laboratory populations because of directional and/or stabilizing selection on phenotypes whose genetics derive from mutation or segregation and recombination of pre-existing diversity. Here we present a model that overcomes some of the existing empirical short-comings in addressing these issues.

We first created a hybrid population of several wild isolates and established an androdioecious population with abundant genetic diversity whose outcrossing rates can vary in response to novel environments. We also created a male-female dioecious population with similar standing diversity to the androdioecious population. From each of these two populations we further derived inbred lines with little diversity. Replicated experimental evolution was then performed in all populations and both sex ratios and male competitive performances measured during 100 generations. If males, through their role in outcrossing, are maintained under androdioecy because they increase the additive genetic variance for fitness, then the following results are expected: 1) male competitive performances are a component of fitness, regardless of male frequencies; 2) male frequencies will be under directional and/or stabilizing selection in androdioecy; 3) higher standing levels of diversity will determine more extensive adaptation than lower diversity levels; and 4) higher competition for fertilization among males under dioecy will lead to more extensive adaptation than under androdioecy.

## Results

### Construction of ancestral populations

We used a funnel cross strategy to derive an androdioecious population with standing genetic diversity from 16 wild isolates (see supplementary materials, **Figure S1** and **Table S1**; see also **Material and Methods**). We then introgressed a sex-determination mutant allele, *fog-2(q71)*, into this population to transform hermaphrodites into females [39], thus creating a dioecious population with obligatory male-female outcrossing. We characterized these two hybrid populations through genotyping 375 SNPs, distributed uniformly across chromosomes IV and X, which corresponds to 35% of the *C. elegans* genome (see **Table S2** for SNP information). Although parental wild isolates come from different collection sites and dates, we confirmed the results of a previous study reporting that only 14 genotypes are present among the 16 wild isolates (not shown; [40]).

**Figure 1:**
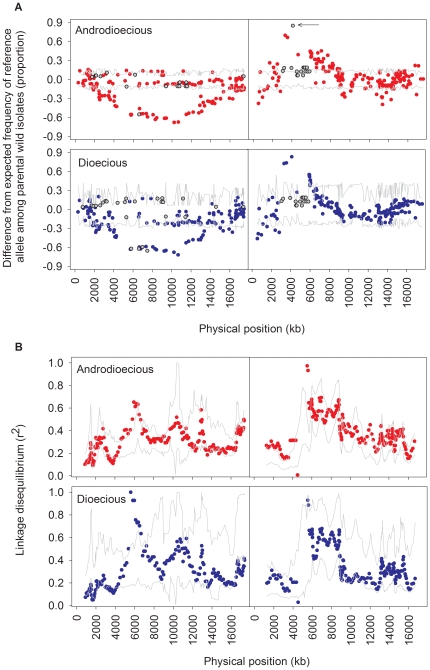
Observed SNP allele frequencies in ancestral populations. In **A** is represented the SNP frequency difference from the expected frequency among parental wild isolates of a reference allele (N2 wild strain) relative to physical position for chromosome IV (left panels), or chromosome X (right panels). Top panels show observed SNP allele frequencies in the ancestral androdioecious population (red). Bottom plots represent observed frequencies in the dioecious population (blue). Gray lines indicate the 95% of simulated data expected under random sampling during the derivation (see **Materials and Methods**). Gray circles represent SNPs that were lost during the derivation, as measured by a minor allele frequency of <0.026 in either population. The arrow indicates the SNP upon which a selective sweep of the wild isolate N2 allele is modeled during the androdioecious derivation. In **B**, linkage disequilibrium among pair-wise SNPs is shown in 10 SNP-wide windows with overlapping step sizes of 1 SNP. Values are plotted at the mean physical position of the SNPs present in each window. Gray lines indicate the 95% of simulations data.

We used simulations to determine the expected SNP allele frequencies with random sampling during the funnel cross and the introgressions (see **Materials and Methods**). Our results revealed that after the derivation the androdioecious population had significant deviations from the expected distributions of SNP allele frequencies (**Figure 1A**). These deviations involved about 50% of the SNPs, independently of the chromosome, and approximately 10% of SNPs loss during the derivation. Likewise, the dioecious population derivation also involved large deviations from expectations and loss of diversity similar to that observed for the androdioecious derivation (**Figure 1A**). Most of the linkage disequilibrium (LD) among pair-wise SNPs achieved after the derivation of both ancestrals was however similar to expected values (**Figure 1B**).

**Figure 2:**
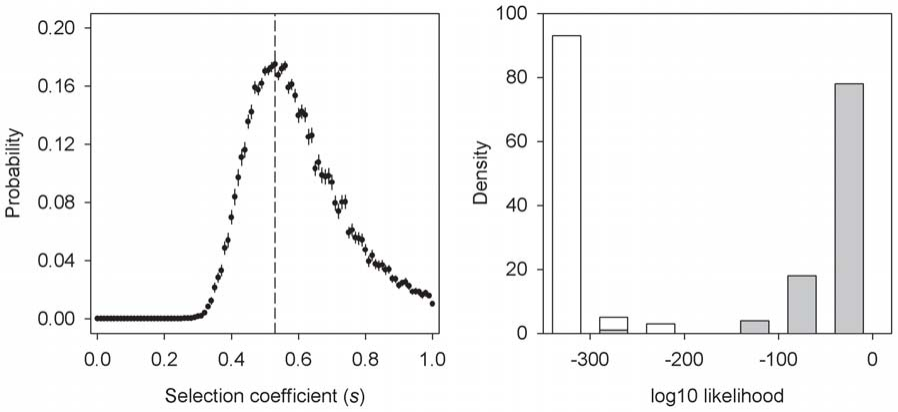
Selection of the N2 wild isolate *pas15121* allele. Left panel shows the probability distribution of having selection coefficients (*s*) explaining the allele frequency changes observed during the derivation of the androdioecious population at marker *pas15121* (see **Materials and Methods**). Error bars indicate the standard error of the mean of the likelihoods of 100 replicate simulations of the funnel cross, with the dashed line indicating the best estimate of *s*. Similarly at the right panel, distributions of *log10* likelihood estimates of allele frequency changes in 19 SNPs, from X-chromosome positions 3,054,487 bp to 5,819,445 bp, for neutral sampling in empty bars, or as grey bars for selection at marker *pas15121* with *s* = 0.53. For each of the 100 simulated replicates compound likelihoods are calculated by multiplying the probability of observed frequencies given the simulated frequencies of each marker.

Significant deviations from random sampling during the derivation could result from selection at particular sites that would promote diversity loss at linked locations by hitch-hiking. To illustrate the extent of this phenomenon, we simulated again the funnel cross with selection at a SNP that showed large frequency changes, and which is located in a 3 Mb region of chromosome X (see arrow in **Figure 1A**; **Material and Methods**). In this region an N2 wild isolate allele almost reached fixation by the time the androdioecious population was established. Results from these simulations with selection indeed showed that coefficients of *s* = 0.53 were sufficient for loss of diversity to occur across the entire region (**Figure 2**).

### Genetic diversity in ancestral populations

**Figure 3:**
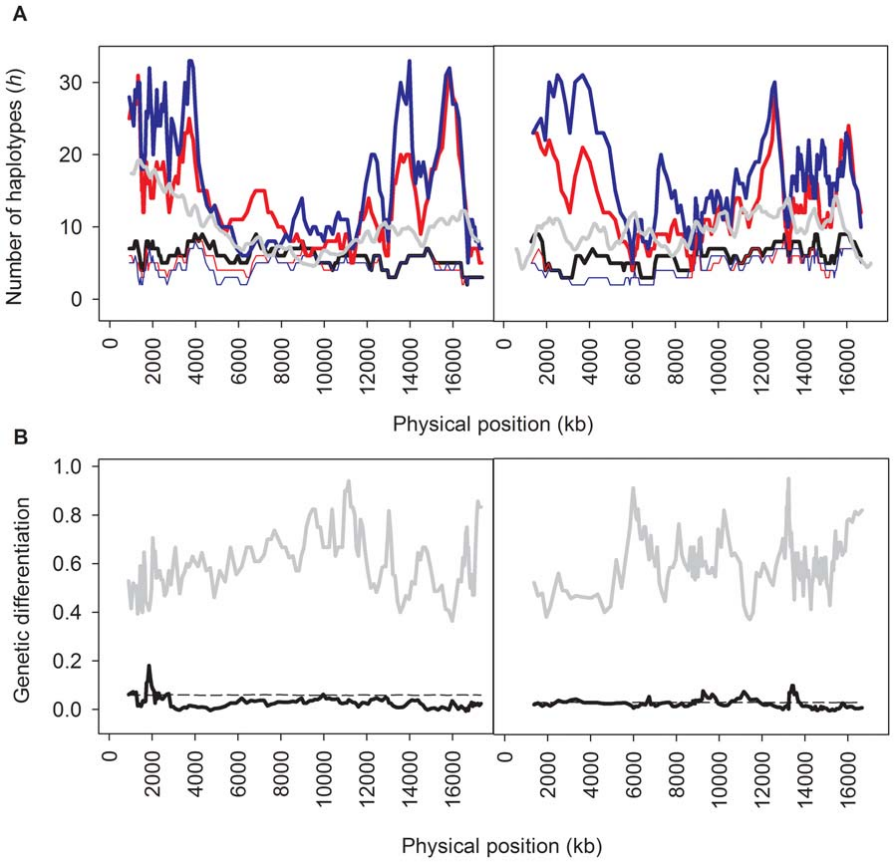
Genetic diversity and differentiation among ancestral populations. **A** shows the number of haplotypes, calculated in 10 SNP windows and step sizes of 1 SNP, along the physical distance (see also **Figure S2**); red = androdioecious population; blue = dioecious population. Black line indicates the number of haplotypes among the 16 wild isolates used as parentals. Grey line shows the diversity found among worldwide collections of *C. elegans* isolates, as recalculated from [40] (see **Materials and Methods**). Thin colored lines show the number of parental isolate haplotypes identified in the two ancestral populations. **B**, the grey line indicates the number of shared haplotypes among the two ancestral populations, shown in 10 SNP-wide windows and step sizes of 1 SNP, as calculated by the proportion of the total number of haplotypes common to both populations. Black lines show the *F_ST_* estimated from these shared haplotypes with their significance, obtained by permutation of SNP position within each chromosome, the latter shown as a dashed line. All positions are haplotype centered.

Despite the observed differences from random sampling, and potential selection during the derivation of both ancestral populations, there was abundant genetic diversity for experimental evolution. Many new alleles were generated during the derivation, as confirmed by the number of haplotypes calculated at several SNP densities (**Figure 3A**, **Figure S2)**. As expected, the generation of diversity was positively correlated with recombination rates. This can be seen, for example, in chromosome IV, where the low recombination rate central region also showed lower diversity than that of the high recombination rate chromosomal arms (**Figure S2**; [40]). Further, the diversity generated during the derivation was such that in both chromosomes the two ancestral populations had more alleles than those found among a worldwide collection of 127 wild isolates (**Figure 3A**) [40]. Despite this, most parental wild isolate genotypes still segregated in the two ancestral populations, with 70% to 80% of the alleles being identified as parentals (**Figure 3A** **)**.

In addition, the two ancestral populations do not appear to be much differentiated among them. Of the total number of alleles in 10 SNP density windows, 40%–100% revealed to be common to the androdioecious and dioecious populations (**Figure 3B**). When differences in the total number of alleles existed, as observed for example on the left arm of chromosome X (**Figure 3A**), they resulted from unique haplotypes that segregated at low frequencies within each population.

### Evolution of sex ratios

**Figure 4:**
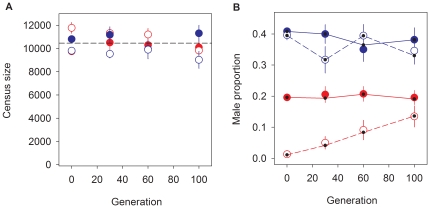
Evolution of sex ratios. In **A**, the census size at the adult stage is shown for dioecious (blue), or androdioecious (red) populations. Empty circles stand for populations without initial diversity; filled circles indicate populations with initial standing diversity. Average values are shown with associated standard error of the mean (SEM) among replicates. Dashed line indicates the overall least-square estimate irrespective of mating system or initial condition of genetic diversity (see **Table 1** for analysis). In **B**, the male proportions measured in all 24 populations (symbols as in **A**). Lines join the least-square estimates by generation at each treatment (dots).

We used 6 replicate populations of each mating system to conduct experimental evolution for 100 consecutive generations. We further derived 6 replicate populations from each mating system via inbreeding of the two ancestral populations to establish an orthogonal treatment with initially low genetic diversity. All 24 populations were contemporaneously measured for total census sizes at the adult stage, after the experiment was over, to confirm that constant densities were followed during experimental evolution (see **Materials and Methods**). Results from these assays confirmed constant census sizes during evolution (**Figure 4A**, **Table 1** for analysis). Thus, one can assume that at each new generation approximately 10^4^ individuals had the opportunity to reproduce, even when accounting for replicate-specific bottlenecks caused by inadvertent culture plate loss during maintenance (log data on plate loss is not shown).

**Table 1:** ANOVA of census size, sex ratio and male competitive performance.

Phenotype	Source	d.f.	Sum-of-Squares	Mean Squares	F	p
Census size	block	2	514498	257249	7.17	<0.001
	generation	3	74506	74506	2.08	0.15
	mating system	1	74506	74506	2.08	0.15
	initial diversity	1	12394	12394	0.35	0.56
	error	147	5276574	35895		
	adj.R^2^ = 6.9%					
Sex ratio	block	2	0.06	0.03	5.62	0.01
	generation (G)	3	0.01	0.00	0.99	0.40
	mating system (M)	1	2.14	2.14	425.17	<0.001
	initial diversity (D)	1	0.23	0.23	45.52	<0.001
	GxM	3	0.05	0.02	2.99	0.03
	GxD	3	0.04	0.01	2.42	0.07
	MxD	1	0.09	0.09	17.97	<0.001
	GxMxD	3	0.04	0.01	2.64	0.05
	error	137	0.69	0.01		
	adj.R^2^ = 78.5%					
Male competitive performance	block	2	0.09	0.04	1.45	0.24
	generation (G)	3	1.86	0.62	20.76	<0.001
	mating system (M)	1	0.01	0.01	0.18	0.68
	initial diversity (D)	1	5.51	5.51	184.13	<0.001
	GxM	3	0.00	0.00	0.00	1.00
	GxD	3	0.33	0.11	3.69	0.01
	MxD	1	0.00	0.00	0.15	0.70
	GxMxD	3	0.12	0.04	1.30	0.27
	error	312	9.33	0.03		
	adj.R^2^ = 44.5%					

Sex ratios were calculated in the same assay plates used to determine census sizes. Our results showed that, irrespective of initial levels of genetic diversity, males from dioecious populations were maintained at average frequencies of 37% (**Figure 4B**). In contrast, the evolution of androdioecious populations was more heterogeneous due to its dependency on initial levels of standing diversity (**Figure 4B**, **Table 1** for analysis). In particular, androdioecious populations with starting diversity stably maintained males at 19.6% throughout the experiment. On the other hand, inbred background populations showed a consistent increase in male numbers, from initial absence to 13.6% by generation 100 (**Figure 4B**). Note that due to high sampling error the sex ratio change in androdioecious populations without initial diversity was only marginally significant (3-way interaction term in **Table 1**; see also **Table S3** for a model accounting for replicate specific population effects).

### Evolution of sex ratio distorters

We next sought to quantify the potential evolution of distorters that could bias the expected sex ratios resulting from outcrossing events.

**Figure 5:**
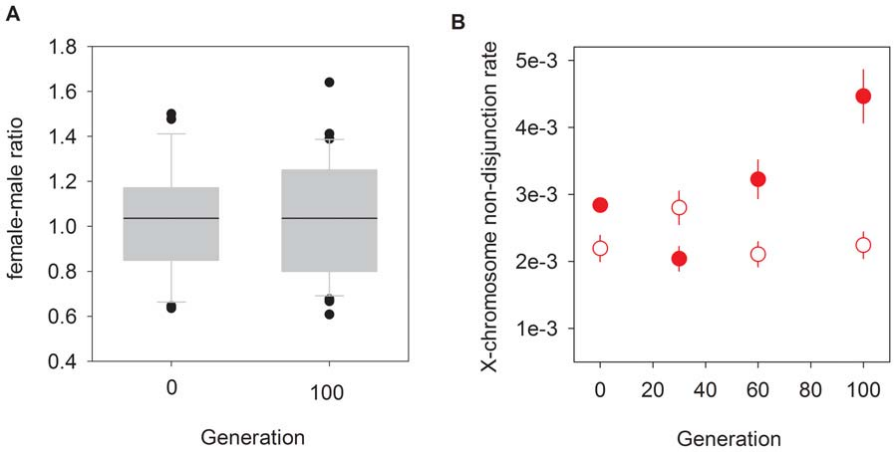
Evolution of sex ratio distorters. **A** shows the offspring sex ratio of individual dioecious males for the G0 population (n = 27) and three G100 replicate populations (total n = 37). Box-plots of the data at 10, 25, 75, and 90 percentiles are represented, along with outliers (dots), and the mean of each generation (line). In **B**, the evolution of X-chromosome non-disjunction rates in androdioecious populations is shown. The proportion of males in the grand-progeny of 50 selfing hermaphrodites was scored in populations with (filled circles) or without (empty circles) starting diversity. Symbols indicate the average number of males per replicate population and mating system with SEM. GLM analysis reveals that generation is significant (p = 0.01) as well as block, initial diversity condition, and interaction (all at p<0.001; total residual deviance d.f. = 325).

For the dioecious populations with standing genetic diversity we estimated the offspring sex ratios from crosses of individual males and standard inbred females (see **Materials and Methods**). The results showed that despite individual variation, at the beginning and end of experimental evolution, mean sex ratios did not deviate from the expected value (**Figure 5A**). There was thus no apparent evolution of sex ratio distorters, as expressed in dioecious males.

For androdioecious populations, with and without initial genetic diversity, we also estimated the rate of X-chromosome non-disjunction (**Figure 5B**). Meiotic X-chromosome non-disjunction is a well known mechanism involved in the generation of *C. elegans* males (males are XØ and hermaphrodites/females XX [4], [34]). Analysis of this data indicates that there was a significant increase of X-chromosome non-disjunction rates with evolution. Evolution was explained by an increase only in those populations with initial diversity, from G30 onwards (**Figure 5B**). X-chromosome non-disjunction was not however sufficient to explain male frequency during experimental evolution, as the numbers produced by this mechanism were minimal.

### Evolution of male competitive performance

**Figure 6:**
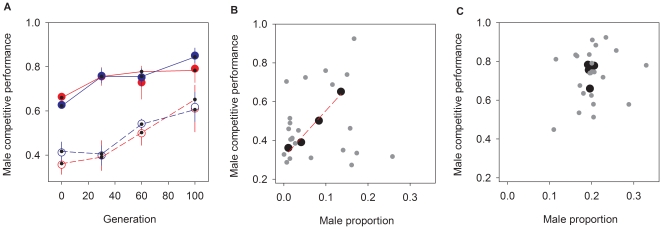
Evolution of male competitive performance. In **A**, the proportion of wild type progeny originated from the competition of experimental males with tester GFP males for the fertilization of standard females (see Materials and Methods). Dioecious populations are shown in blue, androdioecious populations in red. Empty circles show populations without initial genetic diversity and filled circles populations with initial diversity. Circles and bars represent the average values among replicate populations and associated SEM, respectively. Lines connect the least-square estimates (dots) obtained after ANOVA (see **Table 1** for analysis). In **B**, for the androdioecious populations without standing diversity (empty circles) the least-square estimates of male competitive performance (dots in panel **A**) are shown against the least-square estimates of male proportions observed (dots in **Figure 4B**). The correlation among both phenotypes in these populations is positive and significant (*r* = 0.99; *t_2_* = 9.2; *p* = 0.006). In gray the least-square estimates by replicate population at each generation from a fully nested model (**Table S3**). The correlation among these estimates is not significant. In **C**, as in **B**, for androdioecious populations with initial diversity (the correlations among phenotypes are not significant).

As an outcrossing-specific fitness component we measured the experimental male fertilization performance towards standard inbred females, while in competition with tester GFP males (see **Materials and Methods**). Results from these assays clearly revealed that male competitive performance evolved in all populations in a mating system-independent manner (**Figure 6A**; **Table 1** for analysis). Initial genetic diversity condition revealed to be the most significant variable for the evolution of this phenotype: irrespective of mating system, populations with initial standing diversity had high male competitive performances at the beginning of the experiments, while those without initial diversity had much lower male performances (**Figure 6A**). These initial differences continued to be manifested during evolution, as inbred populations not only responded slower until generation 30, but also never attained generation 100 competitive performances comparable to those of diversity-containing populations. Androdioecious populations with initial diversity might have stabilized at high male performances between generations 30 and 60 (see the trajectory of least-square estimates in **Figure 6A**).

### Correlations between male competitive performance and frequencies

We tested for the existence of directional and stabilizing selection on outcrossing by calculating the correlation between male frequencies and male competitive performance during androdioecious experimental evolution (see **Materials and Methods**). The average correlation of evolutionary responses in populations without initial diversity, for both phenotypes, was highly significant (black circles in **Figure 6B**). However, replicate heterogeneity was such that no clear within-population correlation was found (gray circles in **Figure 6B**, see also **Table S3** for analysis). For populations with standing diversity both correlations, between male frequencies and performances, were not significant (**Figure 6C**; **Table S3**).

## Discussion

### Genetically variable populations of *C. elegans* for experimental evolution

To generate genetically diverse populations (in a species which naturally mostly inbreeds), we followed a funnel pair-wise cross strategy among several wild isolates (see [1], [41] for a similar design also in *C. elegans*). As we used large sample sizes we successfully generated a large collection of hybrid genomes (**Figure 3**). Indeed, more diversity was generated than that found in natural populations [40]. Still, more diversity was expected as some was lost during the derivation by strong selection (**Figures 1**, **2**).

That selection would occur during the derivation of the ancestral populations is not surprising. *C. elegans* is known to suffer from outbreeding depression when wild isolates are crossed [11], [12], and such fitness effects should be more pronounced as a higher number of isolates are mixed together and hence a higher number of recombinant deleterious genotypes are produced. We illustrated the presence of large effect fitness loci at a region in chromosome X where loss of diversity in a region as large as 3 Mb surely occurred because of selection. It is in this region that highly pleiotropic RNA expression variants have been mapped between two of the wild isolates used here as parentals [42]. More significantly though is the fact that it is in this region that two major effect loci involved in dispersive and aggregation behaviors are found [15], [43], [44]. In particular, the “solitary" wild type *npr-1* N2 allele almost reached fixation in this region presumably because it confers a benefit in the spatially homogeneous conditions imposed during the derivation, as previously shown [15].

Despite strong selection during the derivation, both the androdioecious and dioecious populations showed similar levels of standing diversity available for experimental evolution (**Figure 3**). We found that most differences must come from the segregation of low frequency alleles. Although these could be important for adaptation, most of the observed responses are expected to come from a large number of fitness loci with intermediate frequency alleles [45]. With similar standing diversity the two ancestral populations with alternative mating systems are thus appropriate to test the consequences of different strengths of selection on outcrossing. Comparison of populations with or without initial diversity evaluated the dependence of selection on mutation over segregation and recombination of pre-existing diversity.

### Sex ratios and the evolution of outcrossing rates

In androdioecious *C. elegans* populations, rates of outcrossing are a function of male numbers due to the inability of hermaphrodites to mate with each other. Consequently, if the sex ratio in the progeny of outcrossing events is one, outcrossing rates equal twice the male frequency [5], [7]. As we showed, although there was evolution of X-chromosome non-disjunction rates, the male number produced by this mechanism cannot bias outcrossing rates estimates (**Figure 5**). We thus estimate an outcrossing rate of 2×0.196 = 0.39 for androdioecious populations with standing diversity, which was stably maintained during experimental evolution. Conversely, those populations starting without diversity eventually reached outcrossing rates of 0.27 after 100 generations (**Figure 4**).

There is however a problem with these calculations. We found that dioecious males segregated at frequencies of 37%, and not the expected 50% value. Further, we determined that the sex ratios of offspring of individual males do not deviated from the expected value of one (**Figure 5**). Thus, we conclude that sex ratio distorters were absent, and that the scored low male number originated from experimental error. On one hand, the observed low male numbers might be related to males being more difficult to identify than females. On the other hand, we noticed that many males fled to the sides of the assay plates and likely desiccated or starved to death, a consistent observation with males being more vagile than hermaphrodites [17]. It is impossible to know if males that escape to the sides of the plates did not reproduce during experimental evolution. But assuming that under androdioecy unaccounted males also reproduced then outcrossing rates are underestimated by a factor of 0.74. As a consequence, a lower bound re-estimate of outcrossing rate for androdioecious populations with standing diversity is of 0.5 throughout evolution. Androdioecious populations without initial diversity should have reached outcrossing rates of 0.34 by generation 100.

It should be noted that our results are similar to those of several previous studies. In particular, the sex ratios observed by Anderson and colleagues [1] are remarkably close to our observations of 20% to 30% in male proportions. Repeatability of this result in different labs is surely due to the common design used in both the construction of the ancestral population (a hybrid of 16 wild isolates was also initially established) and the demographic conditions during experimental evolution (discrete non-overlapping generations at high population sizes were also imposed).

### Outcrossing and adaptation to a novel environment

Males are maintained at much higher frequencies than those expected from mutational input only [5], [7], [26] and thus adaptation must have occurred through a fitness increase in phenotypes exclusive of outcrossing. We first showed this because male competitive performance increased in value during experimental evolution. As expected for a fitness component this occurred independently of mating system or standing levels of diversity (**Figure 6A**, **Table 1**). Secondly, we also confirmed that outcrossing facilitates adaptation because there is a correlation between the average evolutionary responses of male frequencies and male competitive performances, at least in androdioecious populations that depend on mutation for change (**Figure 6B**). And since male competitive performance is a fitness component, directional selection on outcrossing can be inferred. Directional selection was however only found as the average replicate responses during experimental evolution, suggesting that the role of outcrossing on adaptation is highly contingent upon the specific loci that contribute to fitness (**Table S3**). Thirdly, the correlation between an increase in male performances and numbers was not observed in androdioecious populations with standing diversity even though, by definition, they had heritability for outcrossing rates (**Figure 6C**). The stability of sex ratios observed in these populations thus suggests that outcrossing rates were under stabilizing selection and that they maximized fitness under the environmental conditions employed.

Our results are less obvious with regards to the expectation that less initial diversity correlates with less additive genetic variance in fitness, and consequently with constrained adaptation [46]. Up to generation 30, populations lacking initial diversity show male competitive performances that increase slower than those of populations with initial diversity. Additionally, stability of male competitive performance appears to be reached by generation 30 under androdioecy with standing diversity, but not when evolution depends on mutation. However, these results preclude any firm conclusions given the large effects specific to each replicate population (**Figure 6**, **Table S3**). Nevertheless, one can conclude that for periods of up to 100 generations, outcrossing-mediated adaptation was not limited by mutation to a great extent. In initially inbred androdioecious populations, rare males produced by the non-disjunction of the X-chromosome quickly expressed high reproductive success, and thus male numbers increased in frequency with time. Only a few mutations can explain these latter results, even if presumably those have qualitatively much higher fitness effects than the alleles responsible for adaptation from standing diversity (see [47] for estimates of mutation rates and fitness effects during an evolution experiment at high population sizes). Because of this restricted supply of diversity it is not surprising that each replicate population showed highly idiosyncratic dynamics.

Likewise, the expectation that under dioecy there would be increased opportunity for natural and/or sexual selection on outcrossing fitness components was not confirmed (**Figure 6** **;** **Table 1**). This result was not surprising for populations that depend on standing diversity for adaptation, as the additive fitness variance created by outcrossing is expected to be similar among mating systems at the large population sizes employed [48]. Still, this result was surprising for those populations starting evolution without diversity, given that the very large difference in male numbers among mating systems should generate very large differences in the competition for fertilization within each sex. Previous studies that also used experimental evolution from hybrid genomes of wild isolates, have shown that male sperm size increases after 60 generations of evolution under outcrossing vs. selfing conditions [36], [49]. It could be argued that morphological phenotypes such as male sperm size are more sensitive to differences in male numbers than male competitive performance since the latter encompasses a suite of phenotypes involved both in pre- and post-fertilization leading to high environmental variances. Yet, measurements of different phenotypes should not explain our results, as our experimental populations with initial diversity were measured for sperm size as well and neither evolution was observed after 100 generations nor significant differences among mating systems were found (J. Anderson, S. Scholz, and P.C. Phillips, personal communication).

To reconcile both studies we favor the explanation that, in our model system, natural selection for sex-independent outcrossing-specific phenotypes (for example egg-to-adult viability or developmental time), overwhelmed any selection specific to male-male phenotype interactions (cf. [50]). Indeed, our study chiefly confirms previous work suggesting that the fitness variance contributed by all outcrossing phenotypes is increased over the contribution that selfing phenotypes make to fitness. Specifically, Morran and colleagues have observed that the extent of adaptation in experimental populations depends on their mating system: populations with obligatory outcrossing (dioecy) reach higher fitness levels after 40 generations in two novel environments than populations with either facultative outcrossing (androdioecy) or obligatory selfing (monoecy) [2]. A positive correlation between fitness and male frequencies within androdioecious populations was not shown by Morran and colleagues and thus is it possible that higher fitness under dioecy simply resulted from higher opportunity for selection on outcrossing. Given our lack of any significant differences between mating systems though, it is doubtful that under androdioecy evolution only occurred through male phenotypes. Hermaphrodite/female receptivity or propensity to mate [9], [37], and sex-independent phenotypes unique to outcrossing, surely also changed in value because they facilitated adaptation. This issue remains to be addressed however.

The hypothesis that males are maintained because they mitigate inbreeding depression cannot be discarded [5], [24], [25]. Inbreeding depression can only be expressed in populations that segregate genetic diversity. During experimental evolution these populations should suffer from inbreeding depression because they have much higher male competitive performances than populations without initial diversity (which result from several generations of enforced inbreeding). Fitness loss upon inbreeding in a male phenotype is not surprising because, as mentioned above, *C. elegans* suffers outbreeding depression [11], [12] and recombinant recessive deleterious alleles in the X-chromosome that were not lost during the derivation of the ancestral populations can be fully expressed in hemizygous males. But even if inbreeding depression contributed towards male maintenance, it had a minor contribution relative to adaptation. Otherwise differences in the dynamics of male competitive performance would have been observed between mating systems. Particularly, when compared with androdioecious populations, dioecious populations should have been restricted by inbreeding depression as they are expected to have higher effective recombination during experimental evolution [22], [23] (but see [2]).

In conclusion, outcrossing is adaptive under the employed environmental conditions. As a consequence, males are maintained in populations that have the opportunity to reproduce exclusively by selfing. Yet, the fact that males are maintained at intermediate frequencies originates another puzzle requiring investigation: how is selfing maintained within populations that have the possibility to fully reproduce by outcrossing?

## Materials and Methods

### Construction of ancestral populations

16 wild isolates were chosen as parental genotypes of the ancestral populations (see **Table S1** for nomenclature of the strains, and derived populations). Residual heterozygosity in the wild isolates was removed by 10 generations of self-fertilization prior to the beginning of the derivation. After inbreeding, male stocks were obtained from several consecutive crosses of hermaphrodites with rare males produced by the non-disjunction of the X-chromosome.

A funnel cross strategy was used to derive the hybrid androdioecious population (A_0_, see supplementary materials, **Figure S1**). Each of the wild isolates was reciprocally crossed in a pair-wise fashion to create the two-isolate hybrids, which were subsequently intercrossed, also in a pair-wise fashion, to obtain the four-isolate hybrids, and so on, until the final 16-isolate hybrids were obtained. Each hybridization cycle comprised two phases. In the first phase, we generated the reciprocal lineages, with the F1 progeny being sib-mated to produce the F2s, and these again crossed to generate the F3. F3 hermaphrodites were then mated to males from the reciprocal cross, and the resulting F4 progeny intercrossed and mixed in equal numbers. Successful mating was scored by the presence of males in the progeny. Lineages were maintained at high numbers in the second phase of the hybridization cycle (lasting 2 to 4 generations). See **Figure S1** for further details and sample sizes. Once A_0_ was obtained, more than 10^5^ individuals were cryogenically frozen at −80°C.

The hybrid dioecious population was created by the recurrent introgression of the *fog-2(q71)* allele into A_0_. This allele is recessive compared to the wild type, transforming hermaphrodites into females by the disruption of self spermatogenesis, without apparent consequences to males [30]. Specifically, males from strain *JK574*, homozygous for *fog-2(q71)* in a N2 wild isolate background, were mated with 24 A_0_ hermaphrodites, and their offspring allowed to self to generate separate F1s. F2 offspring from each these lineages were scored for *fog-2(q71)* homozygosity by the accumulation of unfertilized oocytes within the body of aging females, and separately kept to initiate another cycle of introgression. There were 9 other such cycles of introgression, starting with the F2 *fog-2(q71)* females being mated with an excess of males from A_0_ (sampled three times from frozen stocks during the whole protocol). The average sample sizes for the first 8 introgression cycles were 53.4±7.8SD females, while the last introgression cycle involved 105.5±2.1SD females. We obtained 199 homozygous *fog-2(q71)* females from this last generation. Each female was then mated to *fog-2(q71)* homozygous males from a different lineage. The derivation of the D_0_ population was concluded after two generations of culturing at high numbers (>10^4^). Based on the genotyping of 77 SNPs at chromosome V, we estimated that about 5 cM surrounding the *fog-2* locus remained to be introgressed, and that the wild type allele segregates in D_0_ with a probability of 10^−4^ given the sample sizes used (data and analysis not shown).

We also derived six populations without starting genetic diversity for each of the two hybrid populations. 11 generations of A_0_ self-fertilization were employed to derive the iA_1–6_ populations, and 22 generations of sib-mating in D_0_ were employed to derive the iD_1–6_ populations. Considering the number of inbreeding generations, a starting homozygosity higher than 98% was expected for both iA_1–6_ and iD_1–6_ [51].

A tester population segregating a GFP (green fluorescent protein) marker was also constructed to measure male competitive performance (see below). The transgenic array *ccls4251(myo3::GFP)* [52] was introgressed from strain *PD4251* into A_0_, following a similar design to that used to derive D_0_, except that only 5 families per introgression cycle were employed.

### DNA collection and genotyping

A_0_ and D_0_ were thawed from samples kept at −80°C, each with >10^3^ individuals, expanded for two generations, and immature L4 hermaphrodites (in the case of the androdioecious population) or females (in the case of the dioecious population) were individually picked. Genomic DNA was prepared with the ZyGEM prepGEMInsect™ kit in a final of water volume of 15 uL. Parental wild isolates were similarly sampled.

447 single nucleotide polymorphisms (SNPs) at chromosomes IV and X were chosen from genome sequence data of the wild isolates N2, CB4856 and CB4858 (**Table S2**; *wormbase* release *WS195*). SNPs were detected by mass determination of allele-specific extension oligonucleotides generated from PCR-amplified genomic DNA using the *iPlex Sequenom™* MALDI-TOF technology platform. For each genotyping assay, 5–10 ng of template DNA was used. Resulting spectra were manually verified and edited with *TYPERv4* by normalizing their intensity with log_10_(height)>0.25 for each SNP allele [53].

After removing monomorphic SNPs within the 16 wild strains (as these might reveal poor assays), data quality control involved excluding SNPs with an incidence of missing data higher than 80%, followed by removing individuals with more than 50% of missing genotypes A third step removed individuals in the upper 5% of the density distribution of missing data. The data set for analysis was composed of 378 SNPs in 89 individuals from the androdioecious population, and 90 individuals from the dioecious population, for a total of 29,972 genotypes in chromosome IV, and 33,442 in chromosome X.

### Simulations of random sampling during the funnel cross and introgression

To determine the expected SNP allele frequency distributions during the construction of the ancestral populations, Monte-Carlo simulations were performed for all SNPs, but separately for each chromosome, by following the crossing and sampling size design represented in **Figure S1**. Custom scripts in R were used to perform the simulations [54].

For the funnel cross, 1000 simulations were done. In each simulation the mating stage was modeled by joining parental gametes (separately at each chromosome) in order to generate the diploid offspring individuals. Each individual then underwent meiosis with a crossover probability of 50% and full homolog-interference, according to a total chromosome size of 50 cM. Crossover position between SNPs was randomly assigned to the chromosomes with probability weights given by the previously described pair-wise genetic distances among SNPs [40]. Chromosomes were modeled as ordered vectors of SNP alleles. The following generation was then created by randomly sampling with replacement segregating chromosomes, assuming a large excess of gametes being produced relative to the number of reproducing individuals. Self-fertilization rate during the second phase of each hybridization cycle was fixed at 0.5, as estimated during experimental evolution (see **Discussion**).

For the dioecious population derivation, 1000 simulations of the introgression cycles of the *fog-2(q71)* allele into the genetic background of A_0_ were performed, following the design and sample sizes described above.

### Selection during funnel cross

To illustrate that selection can explain the clustered loss of diversity at a region of the X-chromosome, maximum likelihood estimates of single locus additive coefficients were first obtained at the SNP marker showing the largest absolute frequency change in the region (*pas15121* SNP located at physical position 4096760 bp; **Table S2**). Selection during the derivation of the ancestral androdioecious population was introduced before mating by attributing to each individual a sampling weight, according to: *w* = 1+*ns*; with *w* being individual fitness, *n* being the number of N2 wild isolate alleles present at the SNP marker, and *s* being a positive coefficient. The likelihood of the observed data was obtained from the binomial probability density distribution (*dbinom* function from *stats* package in R) given the expected allele frequencies with different selection coefficients. A grid of 101 points ranging from 0 to 1, in increments of 0.01, was used for starting *s* values. Likelihood ratio tests were used for significance of the ML estimate against *s* = 0, based on a *Chi*–squared distribution with one degree of freedom. This process was replicated 100 times to obtain a distribution of *s* values that resulted in a final estimate of *s* = 0.53±0.06SD.

To test if selection on a single polymorphism could explain linkage disequilibrium changes, 100 simulations encompassing all fixed SNPs were performed (from position 3,054,487 bp to 5,819,445 bp; n = 19; **Table S2**), assuming *s* = 0.53 on the marker, and including the known genetic distance between SNPs to model recombination [40]. A compound likelihood of observed data for the entire region was calculated by multiplying the likelihoods of each marker, obtained under simulations with and without selection. Given the large differences in the compound likelihood of selection relative to neutrality, further replicate simulations were not required.

### Linkage disequilibrium (LD)

To prevent statistical artifacts markers with minor allele frequencies <0.05 observed in either ancestral population were removed prior to analysis [55]. LD was estimated as composite genotype disequilibria, *Δ*, in that genotype probabilities were the product of the gametic distributions [56]: *r^2^* = *Δ^2^*/*p_i_q_i_p_j_q_j_*, where *p* and *q* designate allele proportions and *i* and *j* the two SNP markers. To prevent any bias in the LD estimators between observed and simulated data, the simulated chromosomes (haplotypes) were converted to un-phased genotypes from where the composite genotype disequilibrium was calculated (see next section).

### Haplotype diversity and differentiation

Haplotype reconstruction from diploid individual data was done with *fastPHASE 1.2* [57], separately for each chromosome. 20 random starts of the EM algorithm were employed with 200 haplotypes taken from the posterior distributions. The number of clusters for the cross-validation procedure was set to 10. SNP positions with posterior probabilities lower than 90% were considered missing data. The number of different haplotypes (*h*) obtained from the phased data was calculated along physical distance in overlapping windows of 2, 5 and 10 SNPs and step size of 1 SNP.

Haplotype number was also estimated at 10 SNP-wide windows and 1 SNP step size from a worldwide collection of 127 wild isolates previously shown to represent 41 distinct genome-wide haplotypes [40]. Since different markers were measured, 1000 subsets from [40] were sampled by jackknifing while matching the density of our data, for a total of 179 SNPs in chromosome IV, and 199 SNPs in chromosome X. Similar estimates were obtained for the 16 wild isolates used here as parentals of the ancestral populations to control the sampling scheme. No differences were detected between the two data sets (results not shown).

Differentiation in shared haplotypes between mating systems, at 10 SNP windows and steps of 1 SNP, was estimated with a multi-allelic version of the *F_ST_* statistic using the *amova* function in the *ade4* R package [58]. Chromosomal significance of differentiation estimates was obtained by finding the 95% upper threshold of *F_ST_* values given with 10000 permutations. In each permutation, the order of markers was reshuffled within each chromosome following the same SNP window density.

### Experimental evolution

Six populations were derived from −80°C stocks and sampling of >10^5^ adult individuals from A_0_ (at a particular generation G#.A_1–6_), and similarly, from D_0_ (G#.D_1–6_). Together with the populations without starting diversity (G#.iA_1–6_; G#.iD_1–6_), all 24 populations were cultured in parallel for 100 generations (cf. [1], [30], [59]). Derived populations were stored at −80°C by G60, being subsequently revived for remaining experimental evolution from large samples (each >10^4^). Each generation started by seeding a synchronized cohort of L1 larval-staged individuals at a density of 1,000 in 9-cm Petri dish plates, for a total of 10 plates per replicate population. Each Petri dish with 28 mL NGM-lite media (US Biological) and with 1 ug of ampicillin supported an *Escherichia coli* lawn (HT115 strain grown O/N to a density of 10^7^–10^8^ cells), used by the worms until passage *ad libitum*. Temperature (20°C) and humidity (80%RH) were kept constant. 3 days later adults were washed out of the 10 plates, mixed with M9 liquid solution in a 15 mL polypropylene tube, and exposed to a 1 M KOH: 5%NaOCl “bleach" solution for 5 min at volumetric ratio of 50∶50. Following 3 rinses with fresh M9 in a new tube (dilution 1∶1,000), the pellet obtained is composed of larval and adult debris, together with fertilized eggs. All centrifugations lasted 30 s at 652rcf. Eggs hatched while in 3–5 mL of M9 in the presence of aeration, and were collected 24 h later at the first larval stage (L1), after removal of dead adults. Appropriate densities of L1s were obtained by scoring live individuals in 5 samples of 5 uL. At each generation the10 Petri dishes of each population were randomized across racks, shelves and incubators. All replicates and experimental treatments were randomized with regards to manipulation and experimenter.

### Census sizes and sex ratios

Samples of A_1–6_, D_1–6_, iA_1–6_ and iD_1–6_, at G0, G30, G60 and G100 were scored for adult density, after being thawed from frozen samples, and expanded in parallel for 2 generations in the same environmental conditions as those of experimental evolution. Measurements were done in three blocks each with two same-numbered replicates and all generation samples. Note that at G0, A_1–6_ and D_1–6_ were pseudo replicates of A_0_ and D_0_, respectively. Two Petri dish plates for each population sample were seeded with 1000 L1 individuals at life-cycle day 1. At day 4, plates were stored at 4°C for 4 to 7 days until scoring. For scoring, each plate was overlaid with a transparent film and individuals sexed and marked with dots under a stereoscope at 7.5× magnification. Films were scanned and analyzed with *ImageJ* software (www.rsbweb.nih.gov/ij/), using standardized thresholds for dot intensity and size. All culturing employed random manipulation, sampling and scoring by treatments. Sex ratios were calculated per assay plate. Quality control of the data included removal of assay plates that had less than 500, or more than 1500, individuals.

Statistical analysis of total census sizes was done by ANOVA (type III sum-of-squares), where generation, mating system and initial diversity were all modeled as fixed factors, while block as a random factor. All interactions were initially modeled, but since none were significant only a reduced model with the main factors is presented. Both data and residuals were normally distributed, as verified with Kolmogorov-Smirnov tests. Sex ratios were similarly analyzed, with the full model being presented. Sex ratio data was not normally distributed (and no transformation of scale was possible) but the residuals of the model presented were normally distributed.

### Offspring sex ratio of dioecious males

To determine the potential evolution of sex ratio distorters in the dioecious populations, males of the D_0_ population (n = 27), or of 3 replicate dioecious populations at G100 (D_4–6_; total n = 37), were individually mated at life-cycle day 3 with one homozygous female from strain *JK574*, *fog-2(q71)*. At day 4 both parental individuals were removed, while their adult offspring sexed 3 days later. Mating took place in 6-cm plates spotted with 4-mm diameter bacterial lawn and 100 uL of 80 uM palmitic acid solution added to the edges (so to avoid individual escape to the sides of the plates). Counting and sexing was done at a 10× magnification under a stereoscope. Chi-squared tests were employed at each generation to test for significance (not shown).

### X-chromosome non-disjunction rate of androdioecious hermaphrodites

For each of the 12 androdioecious populations (A_1–6_, iA_1–6_), synchronously thawed G0, G30, G60, or G100 samples (the grand-progeny of 50 immature L4 hermaphrodites) were counted and sexed in 10 plates, following a similar design as that of census size determination. At G0, A_1–6_ were pseudo-replicates of A_0_. Measurements were performed in 3 blocks. Quality control of the data involved removal of plates where males were not found. Since the occurrence of males is a rare event, statistical analysis on male proportions was done using a generalized linear model (GLM) with a *logit* link function modeling binomial error distributions. We tested for the effects of block, generation, starting diversity and interaction between generation and starting diversity. Mean effects were estimated with iterative weighted least-square procedures. Significance was inferred with *Chi-squared* tests on estimated deviances [60]. The function *glm* in the *stats* R package was employed.

### Male competitive performance

Following a similar design as that of a previous study [4], we measured the performance of experimental males in the fertilization of standard females, when in competition with GFP tester males but otherwise presumed similar genetic diversity as the A_0_ population (see above). Samples of A_1–6_, D_1–6_, iA_1–6_ and iD_1–6_, at G0, G30, G60 and G100 were scored, after being thawed from frozen samples, and expanded in parallel for 2 generations. At G0, A_1–6_ and D_1–6_ were pseudo replicates of A_0_ and D_0_, respectively. Measurements were done in 3 blocks each with two same-numbered replicates, with 4 assay plates in each population sample. At life-cycle day 3, 9 experimental males were transferred to a 6-cm Petri dish plate spotted with a 4 mm-diameter bacterial lawn, together with 9 tester males, and 22 adult *fog-2(q71)* females (strain *JK574*). 24 h later, 20 females were transferred to a new plate, and killed with 30 uL of hypochlorite solution. Plates were incubated for 3 days in the conditions of experimental evolution, and the offspring were scored at 30× magnification under a stereoscope for GFP expression. Quality control of the data included removal of all plates for which less than 50 individuals were scored.

Analysis of male competitive performance was done using ANOVA on the proportion of wild-type genotypes after the competition, as above for census size and sex ratio. Generation, mating system and initial diversity were all modeled as fixed factors, while block as a random factor. All interactions were modeled. Data and residuals are normally distributed.

### Correlations between male competitive performances and frequencies

We estimated the mode of natural selection on male frequencies in androdioecious populations as the correlation of average least-square estimates of male frequencies and competitive performances among generations. Least-square estimates were obtained from the ANOVA models above (**Table 1**). Pearson correlations greater than zero were tested for significance with Student t-tests with 2 degrees of freedom. Separate ANOVAs by genetic diversity treatment were performed in order to obtain least-square estimates for each replicate population by sampled generation. Models tested for a fixed block effect, a random generation nested within block effect, and a random replicate nested within generation effect (**Table S3**).

## Supporting Information

Figure S1 **Derivation of the ancestral androdioecious population.** Funnel pairwise cross of 16 wild isolates, each identified at the parental generation P1 by their haplotype identity and in reference [38] (see also **Table S1**). For each hybridization cycle, creating the 2-, 4-, 8- and 16-isolate hybrids, the first two generations (F1, F2) were separately derived for each reciprocal cross (white cells). The F3 individuals were then crossed between reciprocals to generate the F4 generation (yellow). Remaining generations at each cycle involved expansion to high numbers (orange). Parental lineages are shown in grey. Hermaphrodite numbers during the first phase of each hybridization cycle, or the total number of individuals during the second phase, are indicated. The F3 generation is shown as twice the sample size to indicate the two reciprocal crosses. The final 16-isolate hybrid constituted the ancestral androdioecious population for experimental evolution (red; see **Table S1** for nomenclature).
(TIF)

Figure S2 **Total number of haplotypes in ancestral populations.** Number of haplotypes as obtained by phasing genotype data at 2 SNPs (top), 5 SNPs (middle), or 10 SNPs (bottom), all with overlapping step sizes of 1 SNP, along centered physical position. Red and blue lines show haplotype numbers for the androdioecious and dioecious populations, respectively. Vertical lines indicate the limit of the six recombination rate domains at each chromosome, as defined previously [38]. LA, C, and RA indicates the left arm, center and right arm; with meiotic crossover rates of 7.65 cM/Mb, 1.05 cM/Mb, 3.64 cM/Mb at chromosome IV (left panels), and 3.81 cM/Mb, 1.7 cM/Mb, 5.14 cM/Mb at chromosome X (right panels), respectively. T stands for telomeres, which have very low recombination rates. As the SNP window density increases the majority of diversity generated during the funnel cross and introgression was found in chromosomal domains of higher recombination rates.
(TIF)

Table S1 **Nomenclature of strains and populations.**
(DOCX)

Table S2 **SNP information.**
(DOCX)

Table S3 **Nested ANOVA of sex ratio and male competitive performance under androdioecy.**
(DOC)
