# Supplementary figures and images for: Evolution of Outcrossing in Experimental Populations of Caenorhabditis elegans
Source: PLoS One. 2012 Apr 23;7(4):e35811. doi: 10.1371/journal.pone.0035811 (PMC3335146; doi:10.1371/journal.pone.0035811)

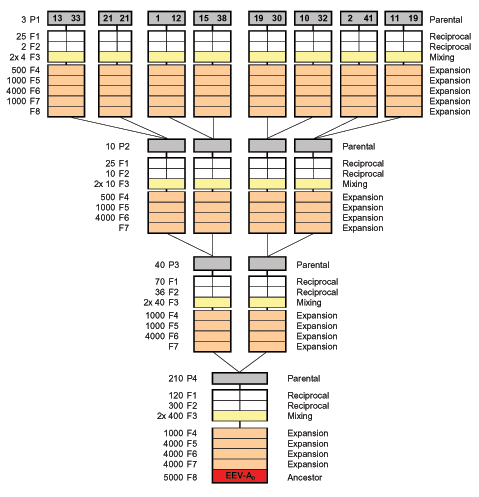

Supplement: Figure S1 — Derivation of the ancestral androdioecious population. Funnel pairwise cross of 16 wild isolates, each identified at the parental generation P1 by their haplotype identity and in reference [38] (see also Table S1). For each hybridization cycle, creating the 2-, 4-, 8- and 16-isolate hybrids, the first two generations (F1, F2) were separately derived for each reciprocal cross (white cells). The F3 individuals were then crossed between reciprocals to generate the F4 generation (yellow). Remaining generations at each cycle involved expansion to high numbers (orange). Parental lineages are shown in grey. Hermaphrodite numbers during the first phase of each hybridization cycle, or the total number of individuals during the second phase, are indicated. The F3 generation is shown as twice the sample size to indicate the two reciprocal crosses. The final 16-isolate hybrid constituted the ancestral androdioecious population for experimental evolution (red; see Table S1 for nomenclature). (TIF) [file pone.0035811.s001.tif]

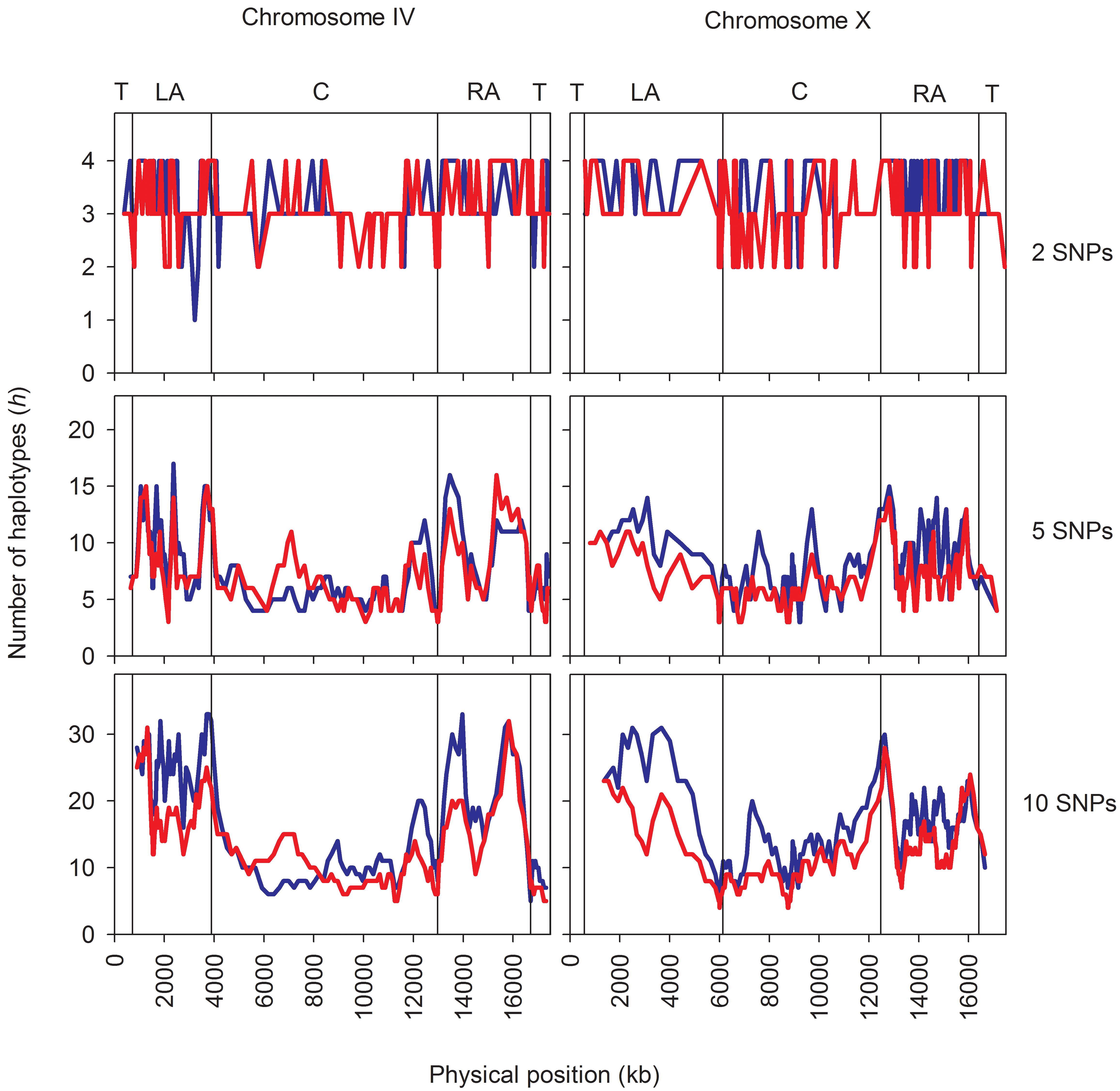

Supplement: Figure S2 — Total number of haplotypes in ancestral populations. Number of haplotypes as obtained by phasing genotype data at 2 SNPs (top), 5 SNPs (middle), or 10 SNPs (bottom), all with overlapping step sizes of 1 SNP, along centered physical position. Red and blue lines show haplotype numbers for the androdioecious and dioecious populations, respectively. Vertical lines indicate the limit of the six recombination rate domains at each chromosome, as defined previously [38]. LA, C, and RA indicates the left arm, center and right arm; with meiotic crossover rates of 7.65 cM/Mb, 1.05 cM/Mb, 3.64 cM/Mb at chromosome IV (left panels), and 3.81 cM/Mb, 1.7 cM/Mb, 5.14 cM/Mb at chromosome X (right panels), respectively. T stands for telomeres, which have very low recombination rates. As the SNP window density increases the majority of diversity generated during the funnel cross and introgression was found in chromosomal domains of higher recombination rates. (TIF) [file pone.0035811.s002.tif]
