# Supplementary material for: Evolution of Outcrossing in Experimental Populations of Caenorhabditis elegans
Source: PLoS One. 2012 Apr 23;7(4):e35811. doi: 10.1371/journal.pone.0035811 (PMC3335146; doi:10.1371/journal.pone.0035811)
Supplement: Table S1 — Nomenclature of strains and populations. (DOCX) [file pone.0035811.s003.docx]

**Table S1. Nomenclature of strains and populations.**

| **CGC_(1)** | **Genotype_(1)** | **Reference** | **Other information** |
| --- | --- | --- | --- |
| AB1 | 13 | CGC, Rockman and Kruglyak (2009) | wild isolate |
| CB4507 | 2 | CGC, Rockman and Kruglyak (2009) | wild isolate |
| CB4852 | 11 | CGC, Rockman and Kruglyak (2009) | wild isolate |
| CB4855 | 21 | CGC, Rockman and Kruglyak (2009) | wild isolate |
| CB4856 | 41 | CGC, Rockman and Kruglyak (2009) | wild isolate |
| CB4858 | 21 | CGC, Rockman and Kruglyak (2009) | wild isolate |
| JU319 | 15 | CGC, Rockman and Kruglyak (2009) | wild isolate |
| JU345 | 32 | CGC, Rockman and Kruglyak (2009) | wild isolate |
| JU400 | 12 | M.-A. Fèlix pers.comm., this study | wild isolate |
| MY1 | 30 | CGC, Rockman and Kruglyak (2009) | wild isolate |
| MY16 | 38 | CGC, Rockman and Kruglyak (2009) | wild isolate |
| N2 | 1 | CGC, Rockman and Kruglyak (2009) | wild isolate |
| PB306 | 33 | CGC, Rockman and Kruglyak (2009) | wild isolate |
| PX174 | 19 | CGC, Rockman and Kruglyak (2009) | wild isolate |
| PX179 | 10 | CGC, Rockman and Kruglyak (2009) | wild isolate |
| RC301 | 19 | CGC, Rockman and Kruglyak (2009) | wild isolate |
| JK574 | 1 | Schedl and Kimble (1988) | Genotype fog-2(q71), N2 background |
| PD4251 | 1 | Fire et al. (1998) | Genotype ccls4251(myo3::GFP), N2 background |
| EEV-A_0_ | - | this study | G0; ancestor androdioecious |
| EEV-D_0_ | - | this study | G0; ancestor dioecious |
| EEV-GFP.A_0_ | - | this study | G0; tester androdioecious |
| EEV-A1 | - | this study | G30, G60, G100; derived replicate |
| EEV-A2 | - | this study | G30, G60, G100; derived replicate |
| EEV-A3 | - | this study | G30, G60, G100; derived replicate |
| EEV-A4 | - | this study | G30, G60, G100; derived replicate |
| EEV-A5 | - | this study | G30, G60, G100; derived replicate |
| EEV-A6 | - | this study | G30, G60, G100; derived replicate |
| EEV-D1 | - | this study | G30, G60, G100; derived replicate |
| EEV-D2 | - | this study | G30, G60, G100; derived replicate |
| EEV-D3 | - | this study | G30, G60, G100; derived replicate |
| EEV-D4 | - | this study | G30, G60, G100; derived replicate |
| EEV-D5 | - | this study | G30, G60, G100; derived replicate |
| EEV-D6 | - | this study | G30, G60, G100; derived replicate |
| EEV-iA1 | - | this study | G0, G30, G60, G100; derived replicate without initial diversity |
| EEV-iA2 | - | this study | G0, G30, G60, G100; derived replicate without initial diversity |
| EEV-iA3 | - | this study | G0, G30, G60, G100; derived replicate without initial diversity |
| EEV-iA4 | - | this study | G0, G30, G60, G100; derived replicate without initial diversity |
| EEV-iA5 | - | this study | G0, G30, G60, G100; derived replicate without initial diversity |
| EEV-iA6 | - | this study | G0, G30, G60, G100; derived replicate without initial diversity |
| EEV-iD1 | - | this study | G0, G30, G60, G100; derived replicate without initial diversity |
| EEV-iD2 | - | this study | G0, G30, G60, G100; derived replicate without initial diversity |
| EEV-iD3 | - | this study | G0, G30, G60, G100; derived replicate without initial diversity |
| EEV-iD4 | - | this study | G0, G30, G60, G100; derived replicate without initial diversity |
| EEV-iD5 | - | this study | G0, G30, G60, G100; derived replicate without initial diversity |
| EEV-iD6 | - | this study | G0, G30, G60, G100; derived replicate without initial diversity |

(1) *Caenorhabditis Genetic Center* (CGC) approved designation

(2) Genotype identity from Rockman and Kruglyak (2009)
