# Supplementary material for: Evolution of Outcrossing in Experimental Populations of Caenorhabditis elegans
Source: PLoS One. 2012 Apr 23;7(4):e35811. doi: 10.1371/journal.pone.0035811 (PMC3335146; doi:10.1371/journal.pone.0035811)
Supplement: Table S2 — SNP information. (DOCX) [file pone.0035811.s004.docx]

**Table S2. SNP information.**

| SNP_(1) | Chromosome | bp_(2) | cM_(3) | Reference allele_(4) | 2nd allele | Reference_genome _(5) | |
| --- | --- | --- | --- | --- | --- | --- | --- |
| pas12042 | IV | 308707 | 0 | A | G | CB4858 | |
| pas1 | IV | 467151 | 0 | G | T | CB4858 | |
| uCE4-507 | IV | 781321 | 0.48958 | A | C | CB4856 | |
| uCE4-511 | IV | 794623 | 0.56549 | G | T | CB4856 | |
| pas2 | IV | 860118 | 0.72063 | T | C | CB4858 | |
| haw50300 | IV | 874853 | 0.79904 | T | C | CB4856 | |
| CE4-228 | IV | 1063709 | 2.5062 | A | G | CB4856 | |
| pas214 | IV | 1160182 | 3.87066 | C | G | CB4858 | |
| uCE4-537 | IV | 1335369 | 6.52981 | G | T | CB4856 | |
| uCE4-540 | IV | 1339309 | 6.52981 | A | C | CB4856 | |
| uCE4-553 | IV | 1445357 | 7.34757 | G | A | CB4856 | |
| uCE4-556 | IV | 1449993 | 7.392 | T | C | CB4856 | |
| uCE4-564 | IV | 1479651 | 7.67627 | C | A | CB4856 | |
| uCE4-566 | IV | 1488728 | 7.76327 | C | A | CB4856 | |
| haw51312 | IV | 1536151 | 8.11557 | T | A | CB4856 | |
| haw51335 | IV | 1548242 | 8.2054 | C | T | CB4856 | |
| uCE4-576 | IV | 1550204 | 8.21997 | A | G | CB4856 | |
| uCE4-580 | IV | 1551262 | 8.22783 | C | - | CB4856 | |
| uCE4-581 | IV | 1556814 | 8.26908 | G | A | CB4856 | |
| uCE4-592 | IV | 1601253 | 8.58257 | G | A | CB4856 | |
| pas12612 | IV | 1685165 | 9.14306 | G | C | CB4858 | |
| uCE4-598 | IV | 1742880 | 9.52856 | T | C | CB4856 | |
| pas12613 | IV | 1745759 | 9.54779 | G | A | CB4858 | |
| uCE4-603 | IV | 1836308 | 10.77468 | G | T | CB4856 | |
| uCE4-606 | IV | 1880589 | 11.44259 | G | T | CB4856 | |
| uCE4-610 | IV | 1919560 | 12.23937 | C | A | CB4856 | |
| haw51978 | IV | 1928856 | 12.42943 | A | C | CB4856 | |
| uCE4-615 | IV | 1959359 | 12.87663 | T | C | CB4856 | |
| CE4-109 | IV | 2095589 | 14.29616 | T | C | CB4856 | |
| haw52330 | IV | 2144755 | 14.63759 | A | G | CB4856 | |
| uCE4-628 | IV | 2161481 | 14.75374 | G | C | CB4856 | |
| haw52443 | IV | 2211333 | 15.66947 | T | A | CB4856 | |
| uCE4-648 | IV | 2212459 | 15.68814 | G | A | CB4856 | |
| uCE4-650 | IV | 2247716 | 16.27285 | A | G | CB4856 | |
| uCE4-668 | IV | 2444713 | 17.70709 | T | C | CB4856 | |
| uCE4-670 | IV | 2457184 | 17.79611 | T | C | CB4856 | |
| haw53023 | IV | 2489588 | 17.79611 | T | C | CB4856 | |
| haw53163 | IV | 2536644 | 18.01662 | G | A | CB4856 | |
| uCE4-677 | IV | 2613045 | 18.55725 | C | T | CB4856 | |
| haw53671 | IV | 2778962 | 19.17343 | A | G | CB4856 | |
| pas13388 | IV | 2894642 | 19.56926 | C | T | CB4858 | |
| uCE4-708 | IV | 3049104 | 20.18391 | C | T | CB4856 | |
| uCE4-716 | IV | 3171602 | 20.73577 | C | T | CB4856 | |
| uCE4-732 | IV | 3269449 | 20.73577 | A | G | CB4856 | |
| uCE4-752 | IV | 3442640 | 21.78067 | T | C | CB4856 | |
| CE4-120 | IV | 3480459 | 21.97101 | A | T | CB4856 | |
| uCE4-763 | IV | 3486826 | 22.01698 | G | T | CB4856 | |
| haw54786 | IV | 3526565 | 22.48225 | T | G | CB4856 | |
| uCE4-780 | IV | 3605101 | 23.26636 | G | A | CB4856 | |
| uCE4-783 | IV | 3613551 | 23.2869 | G | A | CB4856 | |
| uCE4-817 | IV | 3882708 | 24.92578 | G | A | CB4856 | |
| uCE4-832 | IV | 4008342 | 25.74443 | C | T | CB4856 | |
| uCE4-845 | IV | 4060319 | 25.75099 | A | G | CB4856 | |
| uCE4-856 | IV | 4121682 | 25.83146 | C | G | CB4856 | |
| uCE4-874 | IV | 4239704 | 26.08403 | T | C | CB4856 | |
| uCE4-878 | IV | 4279105 | 26.1521 | G | A | CB4856 | |
| pas15099 | IV | 4404252 | 26.76184 | C | A | CB4858 | |
| pas16080 | IV | 5151696 | 28.51761 | T | C | CB4858 | |
| pas16085 | IV | 5293180 | 28.6227 | G | A | CB4858 | |
| pas16087 | IV | 5302593 | 28.62802 | C | G | CB4858 | |
| pas16115 | IV | 5649189 | 28.89673 | T | C | CB4858 | |
| uCE4-920 | IV | 5732323 | 28.98822 | T | C | CB4856 | |
| uCE4-928 | IV | 5781584 | 28.98822 | C | T | CB4856 | |
| uCE4-933 | IV | 5814458 | 28.98822 | A | C | CB4856 | |
| pas17018 | IV | 6183977 | 29.46732 | T | C | CB4858 | |
| pas17021 | IV | 6575933 | 29.79477 | C | A | CB4858 | |
| uCE4-960 | IV | 6601567 | 29.86563 | C | T | CB4856 | |
| pas2157 | IV | 6646382 | 29.94719 | G | C | CB4858 | |
| uCE4-964 | IV | 6647215 | 29.94765 | T | C | CB4856 | |
| CE4-139 | IV | 6790549 | 30.02604 | G | A | CB4856 | |
| uCE4-974 | IV | 6975030 | 30.26717 | G | A | CB4856 | |
| pas17023 | IV | 6983907 | 30.26717 | T | C | CB4858 | |
| CE4-143 | IV | 7341696 | 30.64583 | C | A | CB4856 | |
| uCE4-985 | IV | 7427990 | 30.99618 | A | T | CB4856 | |
| uCE4-987 | IV | 7470605 | 30.99618 | T | C | CB4856 | |
| pas18735 | IV | 7849783 | 31.3965 | C | T | CB4858 | |
| uCE4-1020 | IV | 8046240 | 31.55647 | T | C | CB4856 | |
| uCE4-1023 | IV | 8193378 | 31.83488 | G | T | CB4856 | |
| uCE4-1027 | IV | 8271873 | 31.8772 | G | A | CB4856 | |
| uCE4-1033 | IV | 8393396 | 31.8772 | G | A | CB4856 | |
| uCE4-1045 | IV | 8419277 | 31.8772 | A | T | CB4856 | |
| pas20842 | IV | 8535097 | 31.96657 | C | T | CB4858 | |
| uCE4-1059 | IV | 8569553 | 32.03717 | T | A | CB4856 | |
| pas20865 | IV | 8946994 | 32.56592 | T | A | CB4858 | |
| pas22095 | IV | 9023262 | 32.84577 | T | C | CB4858 | |
| pas22094 | IV | 9123133 | 32.86517 | G | T | CB4858 | |
| uCE4-1071 | IV | 9225944 | 32.92537 | C | G | CB4856 | |
| uCE4-1072 | IV | 9315569 | 32.92537 | G | A | CB4856 | |
| CE4-32 | IV | 9318640 | 32.92537 | C | T | CB4856 | |
| pas22097 | IV | 9390314 | 32.92537 | G | T | CB4858 | |
| uCE4-1075 | IV | 9406524 | 32.92537 | C | T | CB4856 | |
| uCE4-1080 | IV | 9548887 | 32.92537 | A | G | CB4856 | |
| uCE4-1086 | IV | 10073916 | 33.56682 | C | T | CB4856 | |
| uCE4-1094 | IV | 10228846 | 33.56682 | C | T | CB4856 | |
| uCE4-1097 | IV | 10259772 | 33.56682 | T | C | CB4856 | |
| uCE4-1110 | IV | 10279576 | 33.56682 | T | C | CB4856 | |
| pas22976 | IV | 10570562 | 33.84839 | C | T | CB4858 | |
| uCE4-1116 | IV | 10653079 | 33.88993 | C | T | CB4856 | |
| CE4-172 | IV | 10677530 | 33.88993 | A | G | CB4856 | |
| CE4-34 | IV | 10718507 | 33.93135 | A | G | CB4856 | |
| pas22978 | IV | 10769341 | 33.98274 | G | T | CB4858 | |
| pas22979 | IV | 10793215 | 34.00687 | G | A | CB4858 | |
| CE4-174 | IV | 11082972 | 34.29103 | C | G | CB4856 | |
| pas23447 | IV | 11169240 | 34.29103 | G | T | CB4858 | |
| uCE4-1138 | IV | 11247282 | 34.29103 | C | G | CB4856 | |
| pas23449 | IV | 11413248 | 34.29103 | A | C | CB4858 | |
| uCE4-1146 | IV | 11474600 | 34.29103 | G | A | CB4856 | |
| pas4467 | IV | 11477716 | 34.29103 | G | C | CB4858 | |
| pas23450 | IV | 11550350 | 34.46936 | A | G | CB4858 | |
| CE4-178 | IV | 11603947 | 34.69693 | A | G | CB4856 | |
| CE4-1 | IV | 11668234 | 34.69693 | T | C | CB4856 | |
| uCE4-1155 | IV | 11747239 | 34.69693 | T | C | CB4856 | |
| uCE4-1156 | IV | 11804464 | 34.69693 | C | T | CB4856 | |
| uCE4-1171 | IV | 12124862 | 35.33997 | T | C | CB4856 | |
| uCE4-1173 | IV | 12192670 | 35.33997 | G | T | CB4856 | |
| CE4-185 | IV | 12382899 | 35.49994 | G | A | CB4856 | |
| uCE4-1193 | IV | 12771081 | 36.71134 | T | G | CB4856 | |
| uCE4-1194 | IV | 12780085 | 36.71134 | C | T | CB4856 | |
| CE4-28 | IV | 12841508 | 36.79093 | C | T | CB4856 | |
| CE4-191 | IV | 12878429 | 36.92738 | C | G | CB4856 | |
| uCE4-1212 | IV | 12970333 | 37.19204 | G | A | CB4856 | |
| CE4-230 | IV | 12971747 | 37.19204 | G | A | CB4856 | |
| uCE4-1216 | IV | 12985062 | 37.2232 | A | C | CB4856 | |
| uCE4-1226 | IV | 13000000 | 37.25817 | G | A | CB4856 | |
| uCE4-1231 | IV | 13011403 | 37.30156 | G | A | CB4856 | |
| uCE4-1232 | IV | 13022970 | 37.36273 | G | A | CB4856 | |
| uCE4-1238 | IV | 13082494 | 37.67753 | A | G | CB4856 | |
| uCE4-1244 | IV | 13239120 | 37.91867 | T | C | CB4856 | |
| CE4-194 | IV | 13370548 | 38.66436 | T | C | CB4856 | |
| uCE4-1258 | IV | 13706647 | 39.63616 | C | G | CB4856 | |
| uCE4-1264 | IV | 13874168 | 39.93367 | T | C | CB4856 | |
| uCE4-1266 | IV | 13904323 | 40.03491 | T | C | CB4856 | |
| uCE4-1290 | IV | 14158021 | 40.52041 | C | A | CB4856 | |
| pas27670 | IV | 14199814 | 40.64957 | T | G | CB4858 | |
| uCE4-1301 | IV | 14227265 | 40.80422 | G | A | CB4856 | |
| uCE4-1320 | IV | 14335306 | 41.00994 | T | G | CB4856 | |
| pas5916 | IV | 14347377 | 41.0957 | A | G | CB4858 | |
| uCE4-1337 | IV | 14423622 | 41.24951 | A | G | CB4856 | |
| uCE4-1357 | IV | 14556276 | 41.3291 | T | C | CB4856 | |
| uCE4-1361 | IV | 14574271 | 41.3291 | G | A | CB4856 | |
| CE4-204 | IV | 14843110 | 41.64904 | T | A | CB4856 | |
| uCE4-1414 | IV | 15013740 | 41.97214 | T | C | CB4856 | |
| uCE4-1422 | IV | 15020310 | 42.05183 | A | G | CB4856 | |
| uCE4-1425 | IV | 15141339 | 42.56269 | C | T | CB4856 | |
| haw64011 | IV | 15264869 | 43.02421 | G | A | CB4856 | |
| uCE4-1438 | IV | 15324782 | 43.2781 | T | C | CB4856 | |
| uCE4-1500 | IV | 15937100 | 46.91944 | T | G | CB4856 | |
| uCE4-1503 | IV | 15964319 | 47.07758 | G | A | CB4856 | |
| uCE4-1520 | IV | 16006283 | 47.32139 | A | G | CB4856 | |
| uCE4-1526 | IV | 16161127 | 47.72249 | G | A | CB4856 | |
| uCE4-1531 | IV | 16170752 | 47.72249 | G | A | CB4856 | |
| CE4-213 | IV | 16277944 | 48.20799 | T | C | CB4856 | |
| pas29576 | IV | 16563547 | 49.74286 | A | G | CB4858 | |
| pas29606 | IV | 16606982 | 49.84004 | G | T | CB4858 | |
| pas29618 | IV | 16612942 | 49.84004 | C | T | CB4858 | |
| uCE4-1597 | IV | 16622299 | 49.84004 | G | A | CB4856 | |
| uCE4-1604 | IV | 16630843 | 49.84004 | A | G | CB4856 | |
| pas29650 | IV | 16635921 | 49.84004 | A | G | CB4858 | |
| pas29664 | IV | 16669276 | 49.86853 | G | T | CB4858 | |
| pas6616 | IV | 16686301 | 49.88921 | T | C | CB4858 | |
| pas29685 | IV | 16721565 | 49.93204 | A | G | CB4858 | |
| uCE4-1606 | IV | 16777526 | 50 | T | C | CB4856 | |
| pas6624 | IV | 16820486 | 50 | T | G | CB4858 | |
| pas6625 | IV | 16840936 | 50 | C | G | CB4858 | |
| pas29697 | IV | 16867759 | 50 | T | G | CB4858 | |
| uCE4-1617 | IV | 16942653 | 50 | A | T | CB4856 | |
| pas30739 | IV | 17049726 | 50 | G | A | CB4858 | |
| uCE4-1623 | IV | 17152767 | 50 | A | C | CB4856 | |
| pas30700 | IV | 17190436 | 50 | C | T | CB4858 | |
| uCE4-1635 | IV | 17237255 | 50 | A | G | CB4856 | |
| pas6981 | IV | 17284596 | 50 | G | T | CB4858 | |
| CE4-221 | IV | 17297280 | 50 | T | C | CB4856 | |
| uCE4-1641 | IV | 17321314 | 50 | A | G | CB4856 | |
| pas30723 | IV | 17363659 | 50 | T | C | CB4858 | |
| pas30725 | IV | 17368088 | 50 | T | C | CB4858 | |
| CE4-30 | IV | 17407356 | 50.00001 | G | T | CB4856 | |
| pas6985 | IV | 17433033 | 50.00001 | A | G | CB4858 | |
| pas30731 | IV | 17436319 | 50.00001 | A | G | CB4858 | |
| pas30738 | IV | 17473959 | 50.00001 | T | G | CB4858 | |
| CE6-105 | X | 543069 | 0 | T | G | CB4856 | |
| CE6-106 | X | 636851 | 0.22782 | C | T | CB4856 | |
| pas12053 | X | 729101 | 0.30068 | T | G | CB4858 | |
| uCE6-564 | X | 964496 | 0.80132 | A | G | CB4856 | |
| uCE6-572 | X | 1119922 | 0.90798 | T | G | CB4856 | |
| uCE6-597 | X | 1447717 | 1.60609 | C | T | CB4856 | |
| uCE6-628 | X | 1539713 | 2.30325 | G | A | CB4856 | |
| pas12799 | X | 1703819 | 2.70619 | T | G | CB4858 | |
| CE6-113 | X | 2013370 | 3.2168 | G | A | CB4856 | |
| pas14056 | X | 2107765 | 3.41328 | A | G | CB4858 | |
| uCE6-719 | X | 2208029 | 3.87925 | T | G | CB4856 | |
| uCE6-743 | X | 2359118 | 4.36725 | G | A | CB4856 | |
| uCE6-752 | X | 2391888 | 4.51877 | T | G | CB4856 | |
| pas796 | X | 2494927 | 5.28282 | A | T | CB4858 | |
| pas798 | X | 2581249 | 6.15149 | T | A | CB4858 | |
| pas14095 | X | 2657401 | 6.92519 | G | T | CB4858 | |
| CE6-121 | X | 2810040 | 7.37745 | A | G | CB4856 | |
| uCE6-797 | X | 3054487 | 8.40093 | T | A | CB4856 | |
| uCE6-810 | X | 3204763 | 10.06051 | A | C | CB4856 | |
| pas1279 | X | 3304598 | 10.55767 | A | T | CB4858 | |
| pas15094 | X | 3539323 | 10.87937 | A | C | CB4858 | |
| uCE6-844 | X | 3948008 | 13.0769 | T | C | CB4856 | |
| pas15121 | X | 4096760 | 13.69615 | G | T | CB4858 | |
| uCE6-865 | X | 4507511 | 14.89553 | C | T | CB4856 | |
| uCE6-868 | X | 4553058 | 15.05875 | C | A | CB4856 | |
| uCE6-871 | X | 4615754 | 15.23669 | C | T | CB4856 | |
| pas15116 | X | 4645170 | 15.43707 | C | A | CB4858 | |
| uCE6-883 | X | 4949663 | 16.06475 | A | G | CB4856 | |
| uCE6-890 | X | 5153187 | 16.15137 | C | T | CB4856 | |
| uCE6-892 | X | 5184416 | 16.19477 | G | A | CB4856 | |
| uCE6-908 | X | 5243744 | 16.29067 | G | A | CB4856 | |
| uCE6-917 | X | 5355779 | 16.71463 | C | T | CB4856 | |
| uCE6-924 | X | 5390119 | 16.85596 | T | C | CB4856 | |
| uCE6-937 | X | 5555701 | 17.16766 | A | T | CB4856 | |
| CE6-29 | X | 5621435 | 17.41063 | C | G | CB4856 | |
| uCE6-961 | X | 5819445 | 18.09678 | A | T | CB4856 | |
| pas16955 | X | 5896806 | 18.28384 | T | C | CB4858 | |
| pas16968 | X | 5936212 | 18.6703 | C | T | CB4858 | |
| pas16972 | X | 5945414 | 18.76054 | G | T | CB4858 | |
| pas16993 | X | 5971583 | 18.93277 | G | A | CB4858 | |
| pas2151 | X | 5979046 | 18.94943 | A | G | CB4858 | |
| pas17004 | X | 5984794 | 18.96227 | A | G | CB4858 | |
| pas17010 | X | 5996911 | 18.98933 | G | A | CB4858 | |
| pas18233 | X | 6003509 | 19.00407 | C | T | CB4858 | |
| pas18170 | X | 6022248 | 19.09015 | A | T | CB4858 | |
| pas2683 | X | 6028567 | 19.13967 | C | A | CB4858 | |
| pas2701 | X | 6042292 | 19.24721 | G | A | CB4858 | |
| pas18362 | X | 6050919 | 19.3148 | C | A | CB4858 | |
| pas18229 | X | 6367953 | 20.25594 | C | T | CB4858 | |
| haw104267 | X | 6461916 | 20.418 | C | T | CB4856 | |
| pas18395 | X | 6545127 | 20.55399 | G | T | CB4858 | |
| pas18401 | X | 6549617 | 20.56401 | A | G | CB4858 | |
| pas18439 | X | 6618590 | 20.68352 | A | G | CB4858 | |
| pas2770 | X | 6683263 | 20.77652 | G | A | CB4858 | |
| pas2783 | X | 6740763 | 20.85762 | C | T | CB4858 | |
| pas18509 | X | 6743333 | 20.85762 | T | C | CB4858 | |
| pas2790 | X | 6768349 | 20.86919 | A | G | CB4858 | |
| pas2827 | X | 6978203 | 21.24905 | T | A | CB4858 | |
| pas21430 | X | 7043580 | 21.34305 | T | C | CB4858 | |
| pas21055 | X | 7143886 | 21.42774 | T | C | CB4858 | |
| pas21131 | X | 7224545 | 21.42774 | G | A | CB4858 | |
| pas21167 | X | 7251988 | 21.42774 | C | T | CB4858 | |
| pas21189 | X | 7273529 | 21.42774 | G | A | CB4858 | |
| pas3842 | X | 7303206 | 21.42774 | T | C | CB4858 | |
| pas3941 | X | 7531652 | 21.54109 | A | G | CB4858 | |
| pas21676 | X | 7711354 | 21.86619 | C | A | CB4858 | |
| pas4015 | X | 7830871 | 22.36945 | C | T | CB4858 | |
| pas21850 | X | 7896359 | 22.55769 | G | A | CB4858 | |
| uCE6-1056 | X | 7940934 | 22.68581 | A | G | CB4856 | |
| pas22178 | X | 8184229 | 23.65607 | C | T | CB4858 | |
| pas22224 | X | 8206014 | 23.71738 | G | A | CB4858 | |
| pas4116 | X | 8207220 | 23.72078 | G | A | CB4858 | |
| pas22310 | X | 8282058 | 23.83277 | T | C | CB4858 | |
| CE6-157 | X | 8548925 | 24.28953 | A | G | CB4856 | |
| pas22589 | X | 8684616 | 24.28953 | A | C | CB4858 | |
| pas22596 | X | 8693683 | 24.28953 | C | A | CB4858 | |
| pas22608 | X | 8719982 | 24.28953 | C | T | CB4858 | |
| pas22611 | X | 8723696 | 24.28953 | G | A | CB4858 | |
| pas22629 | X | 8751903 | 24.29123 | G | T | CB4858 | |
| pas22635 | X | 8759401 | 24.32306 | A | G | CB4858 | |
| pas22651 | X | 8769530 | 24.36607 | C | A | CB4858 | |
| pas22659 | X | 8777612 | 24.40038 | T | C | CB4858 | |
| pas22682 | X | 8804351 | 24.51391 | T | A | CB4858 | |
| pas22700 | X | 8814989 | 24.55907 | G | A | CB4858 | |
| pas22725 | X | 8855886 | 24.63294 | G | T | CB4858 | |
| pas22740 | X | 8865501 | 24.63294 | T | C | CB4858 | |
| pas4266 | X | 8886061 | 24.63294 | T | C | CB4858 | |
| pas22781 | X | 8930693 | 24.69015 | A | G | CB4858 | |
| uCE6-1102 | X | 8952718 | 24.86075 | G | C | CB4856 | |
| pas4286 | X | 8970532 | 24.91941 | G | A | CB4858 | |
| pas22801 | X | 8990281 | 24.98443 | C | T | CB4858 | |
| pas4436 | X | 9064776 | 25.08857 | G | T | CB4858 | |
| pas23379 | X | 9077163 | 25.08857 | C | T | CB4858 | |
| pas4449 | X | 9090956 | 25.08857 | C | T | CB4858 | |
| pas23171 | X | 9166594 | 25.21765 | T | C | CB4858 | |
| pas4398 | X | 9191900 | 25.2624 | A | G | CB4858 | |
| pas23185 | X | 9204859 | 25.28531 | G | A | CB4858 | |
| pas4404 | X | 9234315 | 25.33739 | G | A | CB4858 | |
| pas23210 | X | 9243204 | 25.35311 | A | C | CB4858 | |
| pas23227 | X | 9267009 | 25.3952 | G | A | CB4858 | |
| pas23239 | X | 9287965 | 25.43197 | G | A | CB4858 | |
| pas23276 | X | 9390702 | 25.43197 | G | T | CB4858 | |
| pas4422 | X | 9479650 | 25.43197 | C | T | CB4858 | |
| pas4770 | X | 10088023 | 26.59984 | G | T | CB4858 | |
| pas24042 | X | 10143896 | 26.64978 | C | T | CB4858 | |
| pas24095 | X | 10219340 | 26.77978 | C | T | CB4858 | |
| pas4663 | X | 10232355 | 26.82654 | A | G | CB4858 | |
| pas24180 | X | 10286821 | 26.92961 | T | G | CB4858 | |
| pas24325 | X | 10466528 | 27.17099 | C | T | CB4858 | |
| pas4741 | X | 10650112 | 27.61306 | G | A | CB4858 | |
| pas24455 | X | 10671005 | 27.64116 | T | C | CB4858 | |
| pas4747 | X | 10693473 | 27.7159 | A | G | CB4858 | |
| pas25296 | X | 11055382 | 28.59522 | A | G | CB4858 | |
| pas25211 | X | 11326006 | 29.06346 | A | G | CB4858 | |
| uCE6-1220 | X | 11474665 | 29.36504 | A | G | CB4856 | |
| uCE6-1224 | X | 11552184 | 29.5487 | G | C | CB4856 | |
| uCE6-1227 | X | 11657275 | 29.93736 | A | G | CB4856 | |
| uCE6-1235 | X | 11854296 | 30.1628 | C | A | CB4856 | |
| pas5045 | X | 11916963 | 30.28612 | A | T | CB4858 | |
| uCE6-1241 | X | 12018310 | 30.35591 | T | A | CB4856 | |
| pas27664 | X | 12142351 | 30.46998 | G | A | CB4858 | |
| CE6-188 | X | 12293106 | 30.81154 | C | A | CB4856 | |
| uCE6-1296 | X | 12763107 | 31.50295 | A | T | CB4856 | |
| uCE6-1304 | X | 12878123 | 31.96301 | T | C | CB4856 | |
| pas6295 | X | 13062788 | 33.6867 | C | T | CB4858 | |
| pas28860 | X | 13070274 | 33.72855 | C | T | CB4858 | |
| pas28953 | X | 13085912 | 33.81597 | G | A | CB4858 | |
| pas6179 | X | 13101020 | 33.90043 | G | A | CB4858 | |
| pas28444 | X | 13106941 | 33.93353 | A | G | CB4858 | |
| pas28458 | X | 13131847 | 34.07277 | T | A | CB4858 | |
| pas28462 | X | 13141555 | 34.12704 | C | T | CB4858 | |
| pas6192 | X | 13161206 | 34.2369 | A | T | CB4858 | |
| pas28496 | X | 13181926 | 34.35273 | C | T | CB4858 | |
| pas6214 | X | 13232245 | 34.63403 | G | A | CB4858 | |
| pas6226 | X | 13275753 | 34.86257 | C | T | CB4858 | |
| pas28560 | X | 13279533 | 34.87945 | A | G | CB4858 | |
| pas28571 | X | 13291067 | 34.93096 | A | G | CB4858 | |
| pas28616 | X | 13329052 | 35.1006 | G | T | CB4858 | |
| pas28641 | X | 13347083 | 35.21454 | A | G | CB4858 | |
| pas28681 | X | 13373869 | 35.45326 | A | G | CB4858 | |
| pas28702 | X | 13409118 | 35.6634 | G | A | CB4858 | |
| pas28718 | X | 13454118 | 35.91358 | T | C | CB4858 | |
| pas28759 | X | 13558906 | 36.89082 | G | A | CB4858 | |
| uCE6-1356 | X | 13646874 | 37.6753 | T | C | CB4856 | |
| pas28877 | X | 13768568 | 38.37308 | T | C | CB4858 | |
| pas6322 | X | 13799769 | 38.46307 | C | T | CB4858 | |
| pas6329 | X | 13822250 | 38.56794 | C | T | CB4858 | |
| pas28930 | X | 13840740 | 38.7 | A | C | CB4858 | |
| pas29001 | X | 13936487 | 39.27384 | G | A | CB4858 | |
| pas6356 | X | 13957385 | 39.36927 | A | G | CB4858 | |
| pas6361 | X | 13975369 | 39.45139 | A | G | CB4858 | |
| pas29922 | X | 14235421 | 40.3269 | G | T | CB4858 | |
| pas29982 | X | 14260854 | 40.50904 | C | T | CB4858 | |
| pas29998 | X | 14278016 | 40.72094 | G | A | CB4858 | |
| pas30037 | X | 14321413 | 40.88039 | A | C | CB4858 | |
| pas6761 | X | 14361347 | 41.02711 | C | A | CB4858 | |
| pas30115 | X | 14410410 | 41.03995 | T | C | CB4858 | |
| pas30131 | X | 14428635 | 41.03995 | A | G | CB4858 | |
| pas30171 | X | 14449003 | 41.13513 | C | T | CB4858 | |
| pas30197 | X | 14464055 | 41.2854 | T | G | CB4858 | |
| pas30218 | X | 14472626 | 41.37402 | G | C | CB4858 | |
| pas30232 | X | 14483286 | 41.48424 | A | G | CB4858 | |
| CE6-206 | X | 14547206 | 42.09239 | G | A | CB4856 | |
| pas30363 | X | 14595187 | 42.09239 | C | T | CB4858 | |
| pas30378 | X | 14612677 | 42.37016 | A | G | CB4858 | |
| pas30430 | X | 14691069 | 42.97829 | T | C | CB4858 | |
| pas30460 | X | 14714123 | 43.10882 | T | C | CB4858 | |
| pas30543 | X | 14805292 | 43.45064 | T | C | CB4858 | |
| pas30551 | X | 14810711 | 43.48285 | G | A | CB4858 | |
| pas30589 | X | 14854960 | 43.74586 | G | A | CB4858 | |
| pas6944 | X | 14890423 | 43.94786 | T | G | CB4858 | |
| pas30641 | X | 14939949 | 44.02475 | A | G | CB4858 | |
| pas33891 | X | 15078313 | 44.44937 | A | G | CB4858 | |
| pas33388 | X | 15101759 | 44.75358 | A | G | CB4858 | |
| uCE6-1528 | X | 15196798 | 45.33232 | G | T | CB4856 | |
| pas8070 | X | 15201902 | 45.34276 | A | C | CB4858 | |
| pas33483 | X | 15217166 | 45.37396 | T | C | CB4858 | |
| pas33513 | X | 15263499 | 45.44568 | G | A | CB4858 | |
| pas33527 | X | 15277321 | 45.44568 | T | C | CB4858 | |
| pas33664 | X | 15410573 | 45.98115 | C | T | CB4858 | |
| pas8126 | X | 15427424 | 46.0097 | A | G | CB4858 | |
| pas33717 | X | 15510085 | 46.29906 | G | A | CB4858 | |
| pas33776 | X | 15580744 | 47.23654 | C | G | CB4858 | |
| pas33782 | X | 15586503 | 47.2503 | A | G | CB4858 | |
| uCE6-1577 | X | 15623571 | 47.33886 | A | T | CB4856 | |
| pas33847 | X | 15654612 | 47.41303 | T | G | CB4858 | |
| uCE6-1585 | X | 15697714 | 47.53674 | T | G | CB4856 | |
| pas33921 | X | 15845012 | 47.66815 | T | C | CB4858 | |
| pas33928 | X | 15849215 | 47.69668 | A | G | CB4858 | |
| pas35084 | X | 16024171 | 48.88446 | T | C | CB4858 | |
| pas35423 | X | 16098505 | 49.07502 | C | T | CB4858 | |
| pas34931 | X | 16104875 | 49.07502 | A | G | CB4858 | |
| pas35085 | X | 16256053 | 49.14078 | G | A | CB4858 | |
| pas35174 | X | 16436793 | 49.73035 | T | C | CB4858 | |
| pas35281 | X | 16739700 | 50 | A | C | CB4858 | |
| CE6-239 | X | 16892057 | 50 | T | C | CB4856 | |
| pas37074 | X | 17100621 | 50 | T | C | CB4858 | |
| pas37130 | X | 17317360 | 50 | T | C | CB4858 | |
| pas37228 | X | 17585505 | 50 | G | A | CB4858 | |
|  | | | | | | |  |

(1) *Wormbase* designation, genome release WS195

(2) Physical position (bp), genome release WS195

(3) Genetic position from Rockman and Kruglyak (2009) or calculated by linear interpolation assuming a total chromosome size of 50cM

(4) Reference allele of N2 wild isolate

(5) *CGC* wild isolate designation containing alternative allele
