# Supplementary material for: Evolution of Outcrossing in Experimental Populations of Caenorhabditis elegans
Source: PLoS One. 2012 Apr 23;7(4):e35811. doi: 10.1371/journal.pone.0035811 (PMC3335146; doi:10.1371/journal.pone.0035811)
Supplement: Table S3 — Nested ANOVA of sex ratio and male competitive performance under androdioecy. (DOC) [file pone.0035811.s005.doc]

**Table S3. Nested ANOVA of sex ratio and male competitive performance under androdioecy.**

| **Treatment** | **Phenotype** | **Source** | **d.f.** | **Sum-of-Squares** | **Mean Squares** | **F** | **p** |
| --- | --- | --- | --- | --- | --- | --- | --- |
| Without initial diversity | Sex ratio | block | 2 | 0.02 | 0.01 | 0.83 | 0.47 |
|  |  | generation | 9 | 0.11 | 0.013 | 1.52 | 0.25 |
|  |  | replicate | 12 | 0.1 | 0.008 | 32.21 | <0.001 |
|  |  | error | 20 | 0.005 | 0.0003 |  |  |
|  |  | adj.R^2^=95.5% |  |  |  |  |  |
|  |  |  |  |  |  |  |  |
|  | Male competitive performance | block | 2 | 0.09 | 0.05 | 0.39 | 0.69 |
|  |  | generation | 9 | 1.28 | 0.14 | 1.18 | 0.39 |
|  |  | replicate | 12 | 1.46 | 0.12 | 4.54 | <0.001 |
|  |  | error | 60 | 1.6 | 0.03 |  |  |
|  |  | adj.R^2^=51.8% |  |  |  |  |  |
|  |  |  |  |  |  |  |  |
| With initial diversity | Sex ratio | block | 2 | 0.005 | 0.002 | 0.79 | 0.48 |
|  |  | generation | 9 | 0.03 | 0.003 | 0.99 | 0.5 |
|  |  | replicate | 11 | 0.04 | 0.003 | 1.67 | 0.2 |
|  |  | error | 12 | 0.02 | 0.002 |  |  |
|  |  | adj.R^2^=23.5% |  |  |  |  |  |
|  |  |  |  |  |  |  |  |
|  | Male competitive performance | block | 2 | 0.02 | 0.008 | 0.17 | 0.85 |
|  |  | generation | 3 | 0.48 | 0.05 | 0.97 | 0.51 |
|  |  | replicate | 12 | 0.67 | 0.06 | 3.65 | 0.001 |
|  |  | error | 52 | 0.79 | 0.02 |  |  |
|  |  | adj.R^2^=43.4% |  |  |  |  |  |
